# Impact of a training course on the quality of malaria diagnosis by microscopy in Angola

**DOI:** 10.1186/1475-2875-13-437

**Published:** 2014-11-18

**Authors:** Sofia Moura, Cláudia Fançony, Clara Mirante, Marcela Neves, Luís Bernardino, Filomeno Fortes, Maria do Rosário Sambo, Miguel Brito

**Affiliations:** Health Research Centre of Angola (CISA), Caxito, Angola; Pediatric Hospital David Bernardino, Luanda, Angola; National Malaria Control Programme, Luanda, Angola; Faculty of Medicine Katyavala Bwila, Benguela, Angola; Lisbon School of Health Technology, Lisbon, Portugal

**Keywords:** Malaria, Training, Angola, Impact, Microscopy, Quality

## Abstract

**Background:**

In Angola, malaria is an endemic disease having a major impact on the economy. The WHO recommends testing for all suspected malaria cases, to avoid the presumptive treatment of this disease. In malaria endemic regions laboratory technicians must be very comfortable with microscopy, the golden standard for malaria diagnosis, to avoid the incorrect diagnosis. The improper use of medication promotes drug resistance and undesirable side effects. The present study aims to assess the impact of a three-day refresher course on the knowledge of technicians, quality of blood smears preparation and accuracy of microscopy malaria diagnosis, using qPCR as reference method.

**Methods:**

This study was implemented in laboratories from three hospitals in different provinces of Angola: Bengo, Benguela and Luanda. In each laboratory samples were collected before and after the training course (slide with thin and thick blood smears, a dried blood spot and a form). The impact of the intervention was evaluated through a written test, the quality of slide preparation and the performance of microscopy.

**Results:**

It was found a significant increase on the written test median score, from 52.5% to 65.0%. A total of 973 slides were analysed to evaluate the quality of thick and thin blood smears. Considering all laboratories there was a significant increase in quality of thick and thin blood smears. To determine the performance of microscopy using qPCR as the reference method we used 1,028 samples. Benguela presented the highest values for specificity, 92.9% and 98.8% pre and post-course, respectively and for sensitivity the best pre-course was Benguela (75.9%) and post-course Luanda (75.0%). However, no significant increase in sensitivity and specificity after the training course was registered in any laboratory analysed.

**Discussion:**

The findings of this study support the need of continuous refresher training for microscopists and other laboratory staff. The laboratories should have a quality control programme to supervise the diagnosis and also to assess the periodicity of new training. However, other variables needed to be considered to have a correct malaria diagnosis, such as adequate equipment and reagents for staining and visualization, good working conditions, motivated and qualified personnel.

## Background

In Angola, malaria affects mostly children under five, and it is estimate that this disease is responsible for 60% of hospital admissions and 35% of mortality at this age group [1, 2]. Malaria misdiagnosis is an important cause of additional morbidity and mortality. The World Health Organization (WHO) guidelines recommend biological confirmation of all suspected malaria patients by microscopy or alternatively by rapid diagnostic tests (RDTs). Presumptive treatment is no longer recommended unless diagnostic tests are not accessible [3]. Accurate malaria diagnosis is essential to a successful and sustainable malaria control as it improves patient care (by reducing the misdiagnosis and therefore inadequate treatment of malaria infections and other febrile diseases), reduces the non-rational drug use that can lead to drug resistance [3–5] and enhances the quality of surveillance, preventing the distortion of statistics, misinformed interventions and economic loss [4–6]. A study carried out in Angola [7] found evidence that health workers resist to laboratory results (mostly negative ones) mainly due to the lack of trust in the performed tests.

Microscopy is the reference standard to diagnose malaria and it allows detection, identification of species and quantification of the parasitaemia [8–10]. WHO highlights that the recommended sensitivity for this method (95% at a parasite density of 100/μl) is achievable in high-quality reference laboratories but hard to establish in laboratories from peripheral hospitals and health centres in malaria-endemic countries [11]. Consequently, the quality of results varies considerably from one lab to another, mainly due to the level of expertise of microscopists, quality of reagents and equipment, employed techniques, workload capacity and inefficient quality control procedures [8, 12]. In turn, limited training and supervision of microscopists often leads to unfamiliarity with the different forms of species other than *Plasmodium falciparum*, inaccurate estimative of parasite density, underreported mixed infections and difficulty in detecting cases with low parasitaemia [13, 14]. However, microscopy can reach high performance standards when manned by competent and motivated individuals, supported by effective training and supervision and adequate supply of reagents and equipment [8]. Studies have been made using short training courses and while some showed an increase in knowledge of staff [15, 16], accuracy of microscopy, quality of blood slide preparation [15, 17] and a reduction in specie misclassification [18] others showed no improvement in the quality of malaria diagnosis [19, 20].

Despite that PCR is mostly used in research scenarios due to its high cost and required laboratorial infrastructure, it is easily reproducible and highly optimized for sensitivity (detecting low parasitaemia cases and accurately differentiating the *Plasmodium* species) being used in surveillance as a performance measurer of microscopy [21–23].

This study, conducted in three health facilities from different provinces of Angola, aimed to evaluate the performance of microscopy on the diagnosis of malaria in children using real time PCR (qPCR) as reference method. Additionally, the impact of a three-day refresher course on the performance of microscopists and, therefore, on the quality of microscopy diagnosis, was evaluated.

## Methods

### Study area

Angola has 18 provinces and the capital city is Luanda. In terms of transmission this country is divided into hyperendemic, mesoendemic with stable transmission and mesoendemic with unstable transmission areas [1]. This study was implemented in three laboratories from hospitals in three different provinces of Angola: Bengo, Benguela and Luanda, all belonging to the mesoendemic area with stable transmission. At each hospital the samples were collected from the emergency paediatric department. This study was developed together with the Angola National Malaria Control Programme (ANMCP) and ethical approval for the study was obtained from the Angolan Ministry of Health Ethics Committee.

### Intervention

The training intervention consisted of a three-day refresher course based on WHO training manual [24] and included lectures and practical hands-on sessions on malaria disease, epidemiological data, cleaning and storage of slides, preparation of thick and thin blood films, Giemsa staining, correct use and care of the microscope, examination of blood films and correct identification of *Plasmodium* species [24]. The training courses were implemented, at the laboratories involved in this study, by one technician from the ANMCP and one expert microscopist from Health Research Centre of Angola (CISA Project) from December 2012 to March 2013. It was given a manual for each participant with a colour atlas of the different *Plasmodium* species, so that they could take notes during the course and use it in their laboratories.

### Slide and sample collection

Each laboratory collected 200 samples before and 200 after the training course. These consisted of a slide with thin and thick blood smears, a dried blood spot (DBS) and a form. After the slide examination the three components were sent to CISA Project. The form had the information about presence or absence of the parasite and in the positive cases the *Plasmodium* species.

### Assessing the impact of the intervention

The impact of the intervention was evaluated through three essential points, namely testing, quality of slide preparation and performance of microscopy.

#### Testing

Before and after the training course it was applied the same written test of multiple choice questions to assess the trainees knowledge.

#### Quality of slide preparation

An expert microscopist evaluated the quality of preparation and staining of the blood films. After visual and microscopical examination, the slides were classified into three categories: good, satisfactory and bad. The criteria for this classification are in Table 1.

**Table 1:** **Blood smears classification criteria (Adapted from Kiggundu** ***et al.*** [15]**)**

Classification	Criteria	
	Thick blood smear	Thin blood smear
**Bad**	- Smear too big (diameter greater than 1 cm) or too small (diameter less than 0.5 cm);	- Smear too big (more than half of the slide) or too small (smaller than 0.5 cm);
	- Smear very close from the edge of the slide (less than 1 cm) which enables the use of immersion oil;	- Smear spread unevenly with patchy distribution, streaky and with many tails (greasy slides or edge of the spreader slide chipped);
	- Very thick smear (fine print cannot be read through it);	- Very thick smear that fine print cannot be read through it or too thin with few red blood cells;
	- Poorly stained (red blood cells are not lysed and parasites have a green, red, or blue colour).	- Many red blood cells lysed and lightly stained cells (red and white blood cells) and parasites.
**Satisfactory**	Smear well-made regarding size and location but moderately stained (red blood cells are partially lysed and parasites are lightly stained).	Smear well-made regarding size and location but moderately stained (red blood cells are partially lysed and parasites are lightly stained).
**Good**	- Smear round in shape with a diameter of approximately 1 cm;	- Smear with the right dimension (half of the slide leaving space for thick blood);
	- Smear at least 1 cm away from the edge of the slide;	- Smear spread evenly (without patchy or streaky distribution);
	- Smear density that fine print can be read through it;	- Smear density that fine print can just be read through it;
	- Smear with all of the red blood cells lysed, and the malaria parasites are well-exposed with a bluish pink coloration.	- Smear with intact red blood cells and pink coloration, intact white blood cells properly stained, and malaria parasites are well-exposed with a bluish pink coloration.

### Performance of microscopy

qPCR was used as the reference method to determine the performance of microscopy in malaria diagnosis. Each slide had a corresponding DBS. For the DNA extraction InstaGene™ Matrix (Bio-Rad^®^) was used and for the qPCR the PrimerDesign™ genesig Kit Genomes used according to manufacturer’s instructions. Each sample was assessed for all *Plasmodium* species: *Plasmodium falciparum*, *Plasmodium vivax*, *Plasmodium ovale* and *Plasmodium malariae*.

### Data management and statistical analysis

All data were entered in a database using Microsoft Office Excel^®^ 2010 and analysed with IBM^®^ SPSS^®^ Statistics 21. Performance of microscopy was defined as sensitivity and specificity, using qPCR as gold standard. To compare these measures, before and after the course, Pearson chi-square and Fisher’s exact test, were applied as appropriate, and to compare the slides quality it was used Pearson chi-square test. The results of the written test were analysed by the Wilcoxon test. The *P* value used as statistically significant was <0.05.

## Results

### Written test

The course attendance was 9, 8 and 11 participants for Bengo, Benguela and Luanda, respectively. Considering all three laboratories, there was a significant (*P* < 0.001) increase on the written test median score, from 52.5% to 65.0% of correct answers. The individual analysis by laboratory also showed an increase in the median score with statistical significance. These results are presented in Table 2.

**Table 2:** **Results of the written test before and after the training course**

Laboratory	n	Pre-course		Post-course		
		Median score (%)	Minimum-maximum (%)	Median score (%)	Minimum-maximum (%)	P value*
Bengo	9	**45.0**	20-55	**65.0**	40-85	0.006
Benguela	8	**57.5**	45-70	**67.5**	55-80	0.038
Luanda	11	**55.0**	20-75	**65.0**	45-75	0.003
**Total**	28	**52.5**	20-75	**65.0**	40-85	<0.001

*Wilcoxon test.

### Quality of thick and thin blood smears

973 slides (475 before and 498 after the course) were examined to evaluate the quality of thick and thin blood smears. In general, thick and thin smears were made in the same slide however 17.7% only had a thick smear. Before the course, Bengo delivered 200 slides of which 116 (58.0%) did not have the thin smear. This percentage decreased to 20.8%, after the course. Benguela delivered all samples with both smears and Luanda had 7.1% and 3.0% of slides without thin smear before and after the course, respectively. Considering all laboratories there was a significant increase in quality of both smears. In Bengo, the thick and thin smears had an increase in quality after the course but only the thin smear was significant (*P* = 0.003). Benguela registered a significant (*P* < 0.001) increase in quality of both smears. Luanda had a non-significant decrease in the quality of both smears. These results are presented in Table 3.

**Table 3:** **Evaluation of the quality of thick and thin blood smears before and after the training course**

Classification	Laboratory	Thick blood smear			Thin blood smear			Slides without thin blood smear	
		Pre-course%	Post-course%	***P***value*	Pre-course%	Post-course%	***P***value*	Pre-course%	Post-course%
	*Bengo*	*n = 200*	*n = 197*		*n = 200*	*n = 197*			
Bad		63.5	55.8	0.102	87.0	75.1	0.003^†^	58.0	20.8
Satisfactory		32.0	41.7		13.0	24.4			
Good		4.5	2.5		0.0	0.5			
	*Benguela*	*n = 107*	*n = 99*		*n = 107*	*n = 99*			
Bad		32.7	9.0	<0.001	74.7	18.2	<0.001	0	0
Satisfactory		44.9	25.3		20.6	31.3			
Good		22.4	65.7		4.7	50.5			
	*Luanda*	*n = 168*	*n = 202*		*n = 168*	*n = 202*			
Bad		64.8	68.9	0.724	79.1	80.2	0.073	7.1	3.0
Satisfactory		29.2	25.7		17.9	12.4			
Good		6.0	5.4		3.0	7.4			
	**Total**	*n = 475*	*n = 498*		*n = 475*	*n = 498*			
Bad		57.0	51.8	0.003	81.5	65.8	<0.001	26.9	9.4
Satisfactory		33.9	31.9		16.4	20.9			
Good		9.1	16.3		2.1	13.3			

*Pearson chi-square.

^†^Due to the small numbers the test was performed using only two categories: bad and satisfactory.

### Performance of microscopy

To determine the performance (sensitivity and specificity) of microscopy taking qPCR as the reference method, 1,028 samples with microscopy results and DBS (516 pre-course and 512 post-course) were used. A total of 396, 240 and 392 samples from Bengo, Benguela and Luanda,were obtained respectively. Of the 1,028 samples, 242 (23.5%) and 289 (28.1%) were positive in qPCR and microscopy, respectively (Table 4).

**Table 4:** **Matched-sample description of the data, comparing microscopy and qPCR**

		Total (n = 1028)		Pre-course (n = 516)		Post-course (n = 512)	
		PCR		PCR		PCR	
		Positive	Negative	Positive	Negative	Positive	Negative
Microscopy	Positive	165*	124	99*	57	66	67
	Negative	77**	662	44**	316	33	346
	Total	242	786	143	373	99	413

*One false *P. vivax* infection in microscopy, which was *P. falciparum* (result from qPCR).

**One false negative in microscopy, which was positive for *P. malariae* (result from qPCR).

Some laboratories increased sensitivity and specificity and others decreased but none of these variances were significant as shown in Table 5. Benguela presented the higher values for specificity, 92.9% and 98.8% pre- and post-course, respectively. And for sensitivity the best pre-course was Benguela (75.9%) and post-course Luanda (75.0%). Regarding species identification, Bengo identified one false *P. vivax* infection (which was not confirmed by qPCR) and one false negative in microscopy, which was positive for *P. malariae* using qPCR.

**Table 5:** **Diagnostic accuracy between microscopy and qPCR**

Laboratory	Sensitivity (%)			Specificity (%)		
	Pre-course	Post-course	***P***value	Pre-course	Post-course	***P***value
Bengo	42.3	57.1	0.375*	75.4	68.9	0.225*
Benguela	75.9	62.5	0.344**	92.9	98.8	0.118**
Luanda	72.9	75.0	0.979*	92.3	91.1	0.878*
Total	68.5	66.7	0.869*	84.7	83.8	0.792*

*Pearson chi-square.

**Fisher’s exact test.

## Discussion

It was presented the impact of a refreshment course, conducted in three health facilities from different provinces of Angola, in the improvement of malaria diagnosis. The effect of the intervention was evaluated by a written test, the quality of slide preparation and the performance of microscopy.

From the analysis of the written test is evident that it has an immediate impact on the workers knowledge, which is consistent with previous studies [15, 16]. In the three laboratories, all technicians were asked to participate in the course, however, a very low participation rate was observed. The major reasons pointed were: schedule incompatibility, lack of financial reward on doing the course and the impossibility of laboratory closure in order to enable the participation of all technicians. The researchers believe that the laboratory staff felt uncomfortable on being evaluated and viewed this study as punitive and, therefore, good cooperation was very hard to accomplish. This reflected in difficulties in collecting all the necessary requests: information about routine diagnosis results by microscopy, thick and thin slides and DBS. It was collected all the three requests in 86.9% of the samples (98.7% in Bengo, 82.0% in Benguela and 77.6% in Luanda).

In Ghana, Bates *et al.* [25] used a system of national supervisors and this was an important step for cooperation of the laboratory staff. However, the worker, responsible to collect the slides and forms was also seen as an outsider. The lack of communication between physicians and the laboratory staff, was also an issue which resulted in the less number of samples gathered before and after the course in Benguela. Nevertheless, Benguela was the one with the best median score after the course and had a significant improvement in the quality of both blood smears.

In the analysis of the quality of blood smear Luanda was the only one that registered a decrease. It could be explained by the different organization of the laboratory after the course; previously the majority of slides were made by one person but after the course all technicians, independently of participation in the course, started to do smears and staining. These findings prevent us from drawing any conclusion about the course impact in the quality of smear and accuracy of malaria diagnosis, for this particular laboratory.

One important aspect, pointed by WHO as crucial for achieving good competence and performance, is the motivation of the workers [8] for which good working conditions as adequate equipment and reagents are needed. The quality of malaria diagnostic, among other aspects, is more likely to be compromised by less motivated laboratory staff and the lack of adequate equipment undermines the laboratory performance [26]. The high percentage of slides lacking the thin blood smear raises concern about the relevance of a correct differentiation of *Plasmodium* species. In the present study, Bengo laboratory performed a thin smear in only 60.5% of the samples mainly due to the lack of methanol.

None of the laboratories registered a significant improvement in performance of malaria diagnosis. This failure may be explained by other factors than the training itself, for example in Benguela the specificity was already high before the training course. Ssekabira *et al.* [20] also found high specificity and sensitivity levels before the training course and after they did not find significant improvement in their study sites. Additionally, it is possible that the Luanda laboratory microscopy results were biased by a possible access of the technicians to previous RDTs results.

A high sensitivity is particularly important because of the risk of untreated cases of malaria, a potential lethal disease, and a high specificity is important for reducing the waste of limited resources, the increase of costs for drugs, unnecessary exposure of patients to the adverse effects of anti-malarial medication, for avoid drug resistance and also for prompting clinicians to look for other causes of illness [6, 15].

The decision to use qPCR as the study standard, instead expert microscopy (golden standard), was made considering the difficulty to make a correct diagnosis using poorly executed blood smears. A bad blood smear preparation can leave artefacts commonly mistaken for malaria parasites, which can lead to false-positive results, and false-negative results can appear if inadequate staining is performed. A bad preparation also increases the time and effort that it takes to read a blood smear [15]. The qPCR has advantages comparing to traditional PCR, being more rapid to perform and to obtain results. The one used in this study is a commercial kit but with a limitation, to the authors’ knowledge there are no published studies comparing this kit with expert microscopy. Therefore validation of this kit is needed.

Another limitation of this study was the Hawthorne effect. Since the laboratory staff were aware they were under observation they may have improved their performance due to an increase of attention and not to the training course.

In conclusion, it was found an improvement in the knowledge and quality of blood smear preparation after the training course and so it is remark, at the laboratories, the importance of a continued programme training for technicians, combined to a quality control programme to assess the periodicity of new courses. Microscopy is the golden standard to malaria diagnosis, but it requires an expert microscopist to obtain high levels of sensitivity, so it is essential to invest in regular training and in the qualification of these professionals. In settings where is possible to implement other methods these could be considered as alternative or complement to microscopy. This study only measured the impact of the training course, but for a correct diagnosis it is important to have other conditions as the necessary equipment and reagents for staining and visualization, good working conditions, motivated and qualified personnel.

## Abbreviations

ANMCP: Angola National Malaria Control Programme
CISA: Health Research Centre of Angola
DBS: Dried blood spot
PCR: Polymerase chain reaction
qPCR: Real time PCR
RDT: Rapid diagnostic test
WHO: World Health Organization.
